# The Magnetisation Process of Bulk Amorphous Alloys: Fe_36+x_Co_36−x_Y_8_B_20_, Where: x = 0, 3, 7, or 12

**DOI:** 10.3390/ma13040846

**Published:** 2020-02-13

**Authors:** Katarzyna Błoch, Marcin Nabiałek, Przemysław Postawa, Andrei Victor Sandu, Agata Śliwa, Bartłomiej Jeż

**Affiliations:** 1 Department of Physics, Faculty of Production Engineering and Materials Technology, Częstochowa University of Technology, Al. Armii Krajowej 19, 42-200 Częstochowa, Poland; bloch.katarzyna@wip.pcz.pl (K.B.); bartek199.91@o2.pl (B.J.); 2 Department of Technology and Automation, Faculty of Mechanical Engineering and Computer Science, Częstochowa University of Technology, Al. Armii Krajowej 21, 42-200 Częstochowa, Poland; postawa@ipp.pcz.pl; 3 Faculty of Materials Science and Engineering, Gheorghe Asachi Technical University of Iasi, Blvd. D. Mangeron 41, 700050 Iasi, Romania; sav@tuiasi.ro; 4 Division of Materials Processing Technology and Computer Techniques in Materials Science, Institute of Engineering Materials and Biomaterials, Silesian University of Technology, Konarskiego St. 18a, 44-100 Gliwice, Poland; agata.sliwa@polsl.pl

**Keywords:** bulk amorphous materials, curie temperature, coercive field, Kronmüllertheory

## Abstract

Amorphous Fe- and Co-based alloys possess so-called soft magnetic properties. Due to the high sensitivity of the magnetisation vector to any inhomogeneities occurring in these alloys, it is possible to assess indirectly structural defects. This paper presents the results of research on the structure and magnetic properties of bulk amorphous alloys with a high content of Fe and Co. The magnetic properties of the produced alloys were tested using a Faraday magnetic balance and a vibrating sample magnetometer (VSM). Analysis of the magnetisation process in the region known as the approach to ferromagnetic saturation was carried out in accordance with Kronmüller’s theorem. Magnetisation in magnetic fields of greater than the effective anisotropy field (Holstein-Primakoff para-process) was also studied. For the studied alloys, it was found that an increase in Fe content causesan increase in saturation magnetisation, and decreases in the values of the coercive field and thespin-wave stiffness parameter, Dspf. A relationship was observed between the width of the amorphous halo and the value of the coercive field. However, no significant links were found between either the presence of structural defects and the properties of these materials, or between the Co content and the value of the coercive field.

## 1. Introduction

Classical metal alloys have a crystalline structure. Fe-based materials with an ordered structure have many advantages, including their capacity to work well as construction materials, and they are relatively cost effective. However, only a small proportion of the crystalline alloys have good magnetic properties; for example, Fe-Si laminations with the Goss structure [1]. In this field, crystalline materials are losing ground in competition with amorphous alloys. Amorphous materials are characterised by an absence of long-range atomic order [2,3,4], which results in their completely different properties. Amorphous alloys based on Fe and Co are characterised by promising soft magnetic properties (e.g., low coercive field value and high saturation magnetisation value) [5,6,7,8]. The absence of crystalline structure makes these materials extremely easy to re-magnetise, which results in their relatively low losses [9,10,11]. Therefore, they are used in components of low-loss transformer cores, amongst other applications [12,13]. Alloys with an amorphous structure have defects of a similar nature to those in crystalline materials. These defects are so-called free volumes (point defects) and quasidislocational dipoles (linear defects) [14]. The presence of these defects affects the process of magnetisation in these materials. This effect is evident in the region known as the approach to ferromagnetic saturation. In this region, the magnetisation process is associated with the rotation of the magnetisation vector around the structural defects. The presence of these defects affects the trajectory of the primary magnetisation curve. Kronmüller, who modified the theory of Brown’s micromagnetism and used it to describe magnetisation in amorphous materials [15,16,17,18], has made the greatest contribution to the description of this process. Magnetisation studies are an indirect way to assess the structure of amorphous alloys based on Fe and Co. The purpose of this work was to determine the magnetisation process for bulk amorphous alloys featuring various proportions of Fe and Co. Additionally, based on the results obtained, the effect was determined of the Fe and Co content on the magnetic properties of the studied alloys.

## 2. Materials and Methods

**Figure 1:**
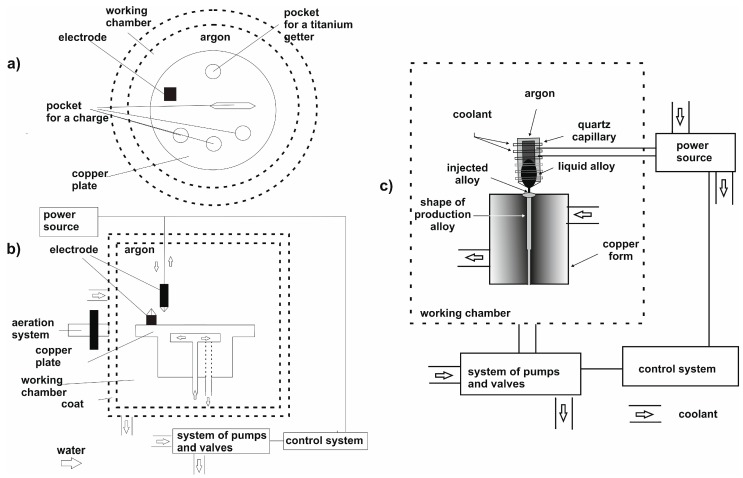
Diagram of an arc furnace: (**a**) Top view, (**b**) front view, (**c**) scheme for producing rapidly-cooled alloys by an injection-casting method.

Polycrystalline alloysamples with the following chemical compositions (at%):Fe_36+x_Co_36−x_Y_8_B_20_ (where: x = 0, 3, 7, or 12) were produced using an arc furnace (Figure 1). Ingots, eachweighing10 g, were made from high-purity ingredients: Fe—99.99%, Co—99.99%, Y—99.95%, B—99.95%. The alloy ingredients were weighed to an accuracy of 0.0001 g. Each alloy composition was melted on a water-cooled copper plate, under a protective argon atmosphere. The melting process was repeated several times; each time, the ingot was physically turned to improve the uniformity of the process. To obtain high ingot purity, a high vacuum (4 × 10^0^ Pa) was created in the working chamber, after which the chamber was flushed with argon and the vacuumre-instated. The solidification process was carried out within an argon atmosphere. Each melting process was preceded by melting a titanium getter.

The polycrystalline ingots were cleaned mechanically, divided into smaller pieces, and then subjected to additional cleaning in an ultrasonic bath. The rapidly-quenched alloy samples were made using an injection-casting process. The polycrystalline load was placed in a quartz capillary with a hole diameter of 1mm; in turn, this capillary assembly was positioned at the top of a copper induction coil. The liquid alloy was melted by eddy-current heating and was forced under argon pressure into awater-cooled copper mould. The alloying process was carried out in the same atmospheric conditions as for the ingots (vacuum level: 4 × 10^0^ Pa, argon pressure in the working chamber: 300 hPa). The scheme for producing rapidly-cooled alloys by an injection-casting method is shown in Figure 1c.

By using an injection-casting method, it is possible to produce alloys at a cooling rate of up to 10^3^ K/s. The resulting samples were cast in the form of rods with the following dimensions: 20mm length, 1mm diameter. The rods were then subjected to further mechanical and ultrasonic cleaning processes. The alloy compositions were selected according to the three criteria formulated by Inoue [19,20]:

- Multi-component alloy;
- Negative mixing heat between the alloying elements;
- Relatively large differences in the lengths of the atomic radii of the alloy components (minimum 12%).

**Figure 2:**
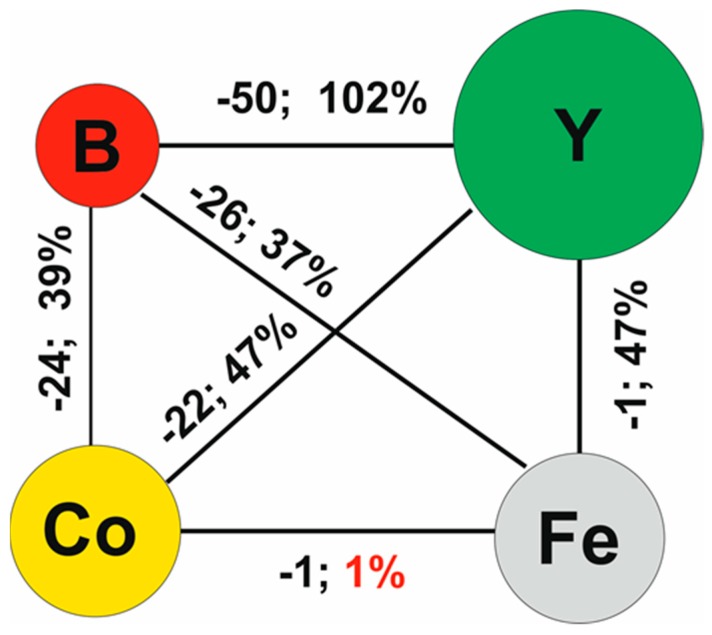
Diagram of mixing heats and the differences in the lengths of atomic radii of the alloying elements.

Figure 2 contains a diagram of mixing heats and the differences in the lengths of atomic radii of the alloying elements.

The studied alloy group meets the criteria of Inoue. Due to the similar properties of Fe and Co, their variable proportions in the alloys should not result in a change in the capacity of each alloy for vitrification. The structure of the alloys was examined using a Bruker D8 Advance X-ray diffractometer. The diffractometer is equipped with a CuKα lamp and a semiconductor meter. X-ray diffraction images were measured over a range of 2Ɵ angle from 30°–100°, with an exposure time of 5s per measuring step (0.02°). Measurements were carried out on samples in the form of powder, without taking into consideration the quantity of powder tested. Thermomagnetic curves were measured, using a Faraday magnetic balance. Magnetic saturation polarisation measurements were carried out over the temperature range from room temperature up to 850 K, in a constant magnetic field of 0.7 T. Primary magnetisation curves and static magnetic hysteresis loops were measured using a vibrating sample magnetometer (VSM) in an external magnetic field of up to 2 T. Magnetic measurements were made for samples in the form of powder. The primary magnetisation curves were analysed according to the Kronmüller theorem. Magnetisation in high magnetic fields can be described by the following equation:

(1) $$\mu_{0}M\left(H\right)=\mu_{0}M_{s}\left[1-\frac{a_{0.5}}{{\left(\mu_{0}H\right)}^{0.5}}-\frac{a_{1}}{{\left(\mu_{0}H\right)}^{1}}-\frac{a_{2}}{{\left(\mu_{0}H\right)}^{2}}\right]+b{\left(\mu_{0}H\right)}^{0.5}$$

where *M_s_*—spontaneous magnetisation; *µ*_0_—magnetic permeability of a vacuum; *H*—magnetic field; *a_i_* (i = 0.5, 1, 2)—angular coefficients of the linear fit, which correspond to the free volume and linear defects; *b*—slope of the linear fit corresponding to the thermally-induced suppression of spin-waves by a magnetic field of high intensity.

The coefficients *a*_0.5_, *a*_1_, *a*_2_, and *b* can be determinedbased on primary magnetisation curve analysis. These coefficients are related to the magnetising process by the following equations:

(2) $$\frac{a_{0.5}}{{\left(\mu_{0}H\right)}^{0.5}}=\mu_{0}\frac{3}{20A_{ex}}{\left(\frac{1+r}{1-r}\right)}^{2}G^{2}\lambda_{s}^{2}{\left(\Delta V\right)}^{2}N{\left(\frac{2A_{ex}}{\mu_{0}M_{s}}\right)}^{0.5}\frac{1}{{\left(\mu_{0}H\right)}^{0.5}}$$

(3) $$\frac{a_{1}}{\mu_{0}H}=1.1\mu_{0}\frac{G^{2}\lambda_{s}^{2}}{{\left(1-\nu \right)}^{2}}\frac{Nb_{eff}}{M_{s}A_{ex}}D_{dip}^{2}\frac{1}{\mu_{0}H}$$

(4) $$\frac{a_{2}}{\mu_{0}H^{2}}=0.456\mu_{0}\frac{G^{2}\lambda_{s}^{2}}{{\left(1-\nu \right)}^{2}}\frac{Nb_{eff}}{M_{s}^{2}}D_{dip}^{2}\frac{1}{{\left(\mu_{0}H\right)}^{2}}$$

(5) $$b=3.54g\mu_{0}\mu_{B}{\left(\frac{1}{4\pi D_{spf}}\right)}^{1.5}kT{\left(g\mu_{B}\right)}^{0.5}$$

where Δ*V*—the change in volume due to the presence of a point defect characterised by a bulk density of *N*, *A_ex_*—exchange constant, *G*—transverse elastic shear modulus, *r*—Poisson’s ratio, *λs*—magnetostriction constant, *k*—Boltzmann’s constant, *µ_B_*—Bohr magneton, *g*—gyromagnetic factor.

Equation (2) describes the effect of the presence of point defects on the magnetisation process. Equations (3) and (4) relate to linear defects that meet the relationships: *D_dip_* < *l_H_* and *D_dip_* > *1_H_*, respectively (where l_H_—exchange distance, *D_dip_*—quasidislocational dipole width). Coefficient b is related to the spin-wave stiffness parameter *D_spf_*.

Based on parameters derived from analysis of the primary magnetisation curve, the exchange constant can be determined:

(6) $$A_{ex}=\frac{M_{s}D_{spf}}{2g\mu_{B}}$$

## 3. Results

**Figure 3:**
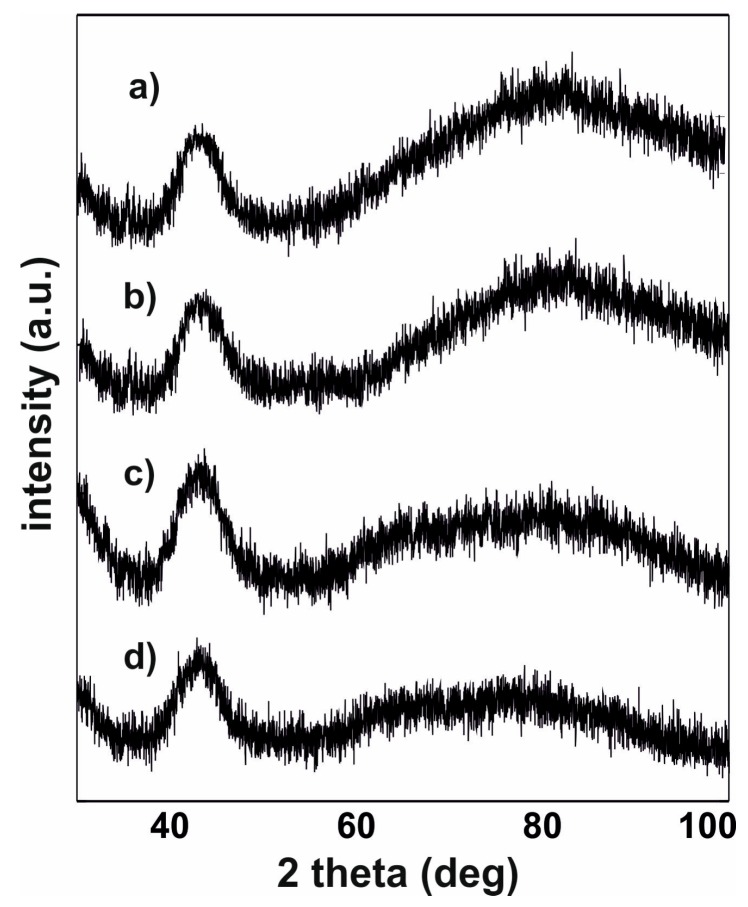
X-ray diffraction images for the alloys: (**a**) Fe_36_Co_36_Y_8_B_20_, (**b**) Fe_39_Co_33_Y_8_B_20_, (**c**) Fe_43_Co_29_Y_8_B_20_, (**d**) Fe_48_Co_24_Y_8_B_20_.

Figure 3 contains X-ray diffraction images measured for the studied alloys.

The resulting data are presented in Table 1.

**Figure 4:**
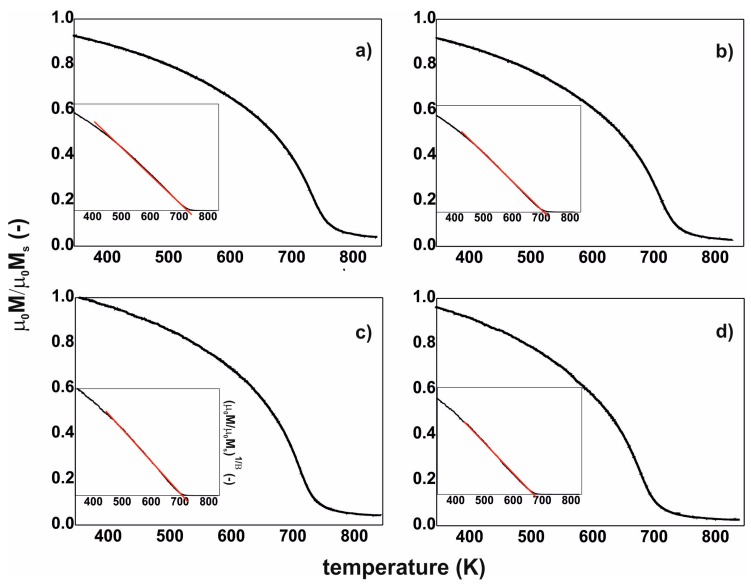
Magnetic saturation polarisation curves, as a function of temperature: (**a**) Fe_36_Co_36_Y_8_B_20_, (**b**) Fe_39_Co_33_Y_8_B_20_, (**c**) Fe_43_Co_29_Y_8_B_20_, (**d**) Fe_48_Co_24_Y_8_B_20_.

Figure 4 contains magnetic polarisation curves as a function of temperature. For ferromagnetic materials that meet Heisenberg’s assumptions, it is possible to determine the Curie temperature using a critical factor β = 0.36 (inserts shown in Figure 4).

**Figure 5:**
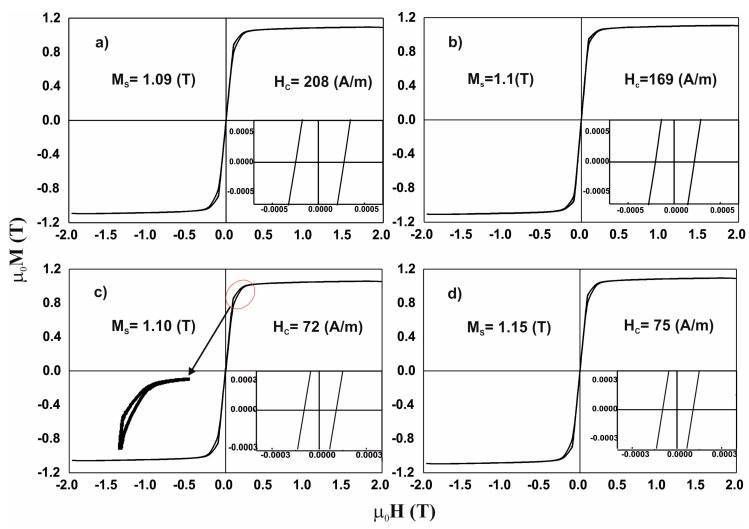
Static magnetic hysteresis loops for the alloys: (**a**) Fe_36_Co_36_Y_8_B_20_, (**b**) Fe_39_Co_33_Y_8_B_20_, (**c**) Fe_43_Co_29_Y_8_B_20_, (**d**) Fe_48_Co_24_Y_8_B_20_.

The determined Curie temperatures are given in Table 2. Figure 5 contains static magnetic hysteresis loops for the investigated alloys.

**Figure 6:**
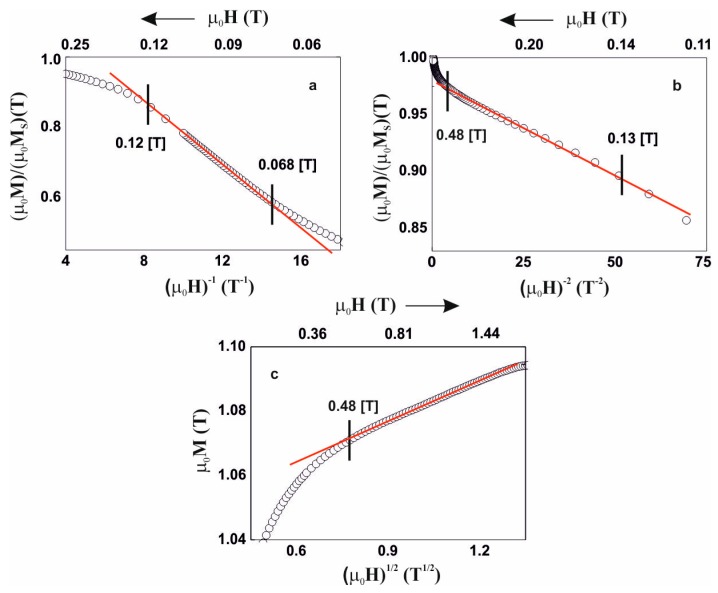
Magnetisation for the alloy Fe_36_Co_36_Y_8_B_20,_ as a function of: (**a**) (µ_0_H)^−1^, (**b**) (µ_0_H)^×2^, (**c**) (µ_0_H)^1/2^.

**Figure 7:**
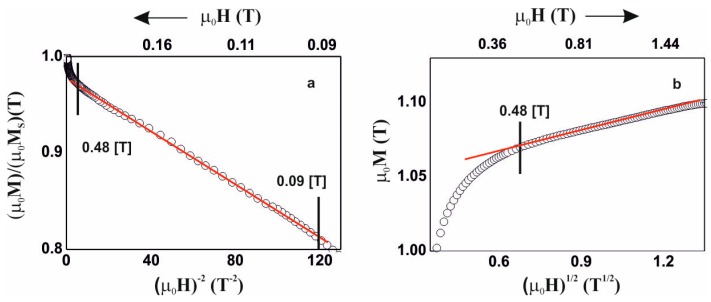
Magnetisation for the alloy Fe_39_Co_33_Y_8_B_20,_ as a function of: (**a**) (µ_0_H)^−2^, (**b**) (µ_0_H)^1/2^.

**Figure 8:**
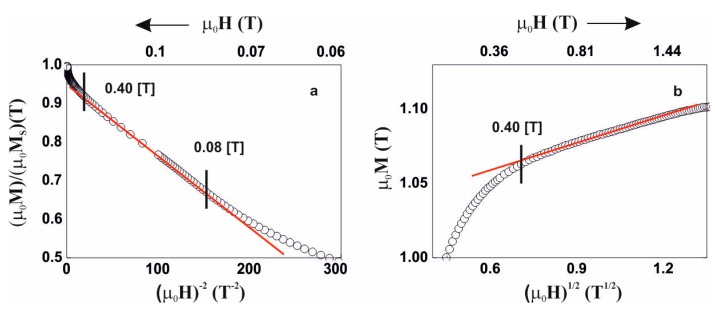
Magnetisation for the alloy Fe_43_Co_29_Y_8_B_20,_ as a function of: (**a**) (µ_0_H)^−2^, (**b**) (µ_0_H)^1/2^.

**Figure 9:**
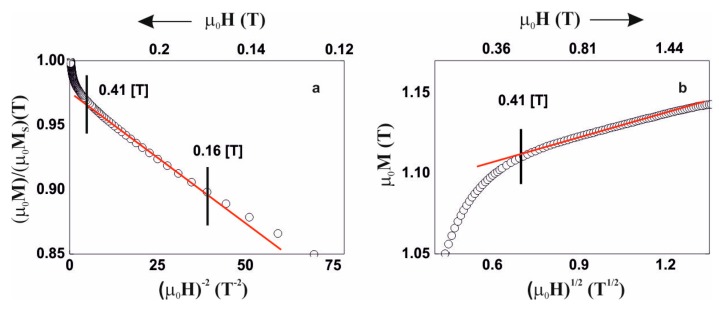
Magnetisation for the alloy Fe_48_Co_24_Y_8_B_20,_ as a function of: (**a**) (µ_0_H)^−2^, (**b**) (µ_0_H)^1/2^.

Due to the above, analysis of the magnetisation process was carried out, in accordance with the theory of Kronmüller, in the area called the approach to ferromagnetic saturation and above this area (so-called Holstein-Primakoff para-process). Analysis of the primary magnetisationcurves is presented in Figure 6, Figure 7, Figure 8 and Figure 9.

The magnetisation process of the Fe_36_Co_36_Y_8_B_20_ alloy is associated with the rotation of the magnetisation vector around linear defects, fulfilling relationships (3) and (4). In the case of the Fe_39_Co_33_Y_8_B_20_ alloy, in the magnetic field range 0.09–0.48 T, the magnetisation process is associated with the presence of quasidislocational dipoles, satisfying relationship (3). Magnetisation of the Fe_36_Co_36_Y_8_B_20_ and Fe_39_Co_33_Y_8_B_20_ alloys in a magnetic field greater than 0.48 T is associated with the damping of thermally-induced spin-waves.

The magnetisation process of Fe_43_Co_29_Y_8_B_20_ and Fe_48_Co_24_Y_8_B_20_ alloys is similar. The process of magnetisation in the area called the approach to ferromagnetic saturation is associated with the presence of linear defects with dimensions not exceeding the exchange distance. Dataderived from analysis of the magnetisation process are presented in Table 2. Based on the determined parameters, the values of the spin-wave stiffness parameter, D_spf_, and the exchange distance, A_ex_, were calculated.

## 4. Discussion

All recorded X-ray diffraction images are typical for materials with an amorphous structure. Only wide fuzzy maxima in the 2Ɵ angle range 40°–50° are visible on the diffractograms. An analysis of these maxima was carried out, their intensity and width were determined.Due to the different quantities of materials applied to the measuring plates, the change in the intensity of the maxima is a direct result of a smaller number of counts per sample. Some dependencies of the maxima shape on the magnetic properties of the samples were noted, and these are described later in this work.

The measured thermomagnetic curves have an individual inflection, associated with the transition of the material from the ferro- to the para-magnetic state. This transition arises from the presence of an amorphous matrix (Figure 3). During measurement, the magnetisation of the alloy does not drop to zero, which indicates the presence of trace quantities of one or more additional magnetic phases within the sample volume. Such a phase may be a crystalline phase with a Curie temperature outside the current measuring range (for example, phase αFe: T_C_=1043 K or phase Fe_2_B: T_C_ = 1015 K). It is also possible that areas with a different chemical composition are present in the amorphous matrix (characterised by ordering—in a similar way to crystalline phases) and these are responsible for an increased value of magnetisation at high temperatures.

All of the measured loops are typical of materials with soft magnetic properties. The tested alloys are characterised by relatively high saturation magnetisation and low coercive field. The loops have an axial shape [21] (see inset in Figure 5c). In the case of the described hysteresis loops, the emergence of the so-called “Ewing knee” is indicative of the presence of magnetic phases with different properties, in the volume of the alloy. The narrow hysteresis loop at the origin of the M-H (magnetisation—magnetic field strength) system, is connected with the soft magnetic properties of the alloys; and the wider parts of the hysteresis loops, presented in the inserts, are connected with the residual presence of magnetically-hard phases. This indicates the residual presence of ordering that hinders the process of magnetisation in high magnetic fields. The process of magnetisation in strong magnetic fields in the third magnetisation area is associated with wall movements of the closing domains and spontaneous magnetisation rotations. In the case of amorphous materials, the shape of the static magnetic hysteresis loop is related directly with chemical and topological disorder, local structural stress, and magnetic heterogeneity. The contribution of local stress within the structure plays the dominant role in the process of high-field magnetisation. The values of the coercive field and saturation magnetisation are associated with short-range stresses. These stresses constitute the so-called free volumes in the material. The presence of free volumes in amorphous samples is related directly to the sample production process and the method of solidification. The increased melt viscosity during rapid solidification promotes the attraction of gas particles and entrapment of them within the melt volume. Within these heterogeneities of the structure, local stresses occur. Based on analysis of the magnetic hysteresis loop, the surface area of the regions—marked in Figure 5—was calculated. The region marked is within the range of the third magnetisation area. For the first of the tested alloys, the coercive field value is 208 A/m and this is the highest of all of the investigated samples. After adding more Fe content to the sample, a decrease in the coercive field value (Fe_39_Co_33_Y_8_B_20_ 169 A/m) was observed. It should be noted that the surface area (marked in Figure 5) for the Fe_36_Co_36_Y_8_B_20_alloy sample is slightly lower than those for the other alloys. In addition, it was found that the introduction of a higher Fe content has a relaxing effect on the magnetic structure of the samples. It is possible to classify the studied alloys by the value of the coercive field and by the extent of the impact of structural defects on the magnetisation process. In the case of the first two alloys tested, with content of Fe,Fe_36_Co_36_Y_8_B_20_ and Fe_39_Co_33_Y_8_B_20_, the impact of heterogeneity of the structure in the form of short-range stresses ends for the magnetic field strength of 0.48 T. The other two alloys (Fe_43_Co_29_Y_8_B_20_ and Fe_48_Co_24_Y_8_B_20_) show a coercive field value that is less than half, and the scope of the effect of free volumes ends at the magnetic field strength of 0.40–0.41 T. It has also been noted that the magnetisation process itself is associated mainly with linear defects. It is evident that increasing the Fe content improves the soft magnetic properties of alloys.

**Figure 10:**
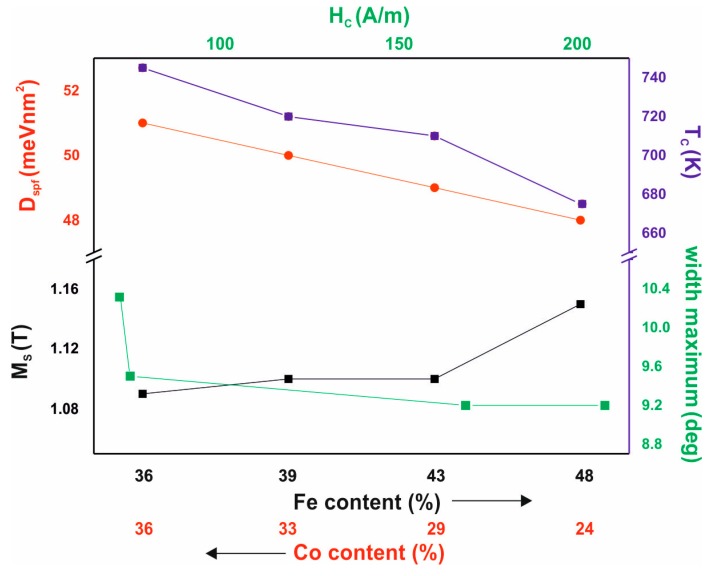
Dependence of magnetic properties on the proportions of Fe and Co and the relationship of the coercive field with the width of the amorphous halo.

Several relationships were observed between the chemical composition and the magnetic properties of the investigated alloys. These relationships are shown in Figure 10.

The studied alloys have very high T_C_ values, and this feature is associated with a significant Co-content. An increase in Fe content—at the expense of Co—causes a decrease in the T_C_ value, an increase in the M_S_ value, and a decrease in the D_spf_ parameter value. The decreasing D_spf_ value, with increasing M_S_ value, is believed to be a particularly interesting phenomenon. The D_spf_ parameter is related to the distance between the magnetic atoms within the alloy and the number of “magnetic atom neighbours” (magnetic atoms have between eight and 12 neighbours [22] depending on the degree of relaxation). Typically, this behaviour can be associated with the presence of antiferromagnetic ordering in certain areas of the sample. In this case, the magnetic atoms may be so close together that antiferromagnetic order may be more privileged in energy terms. No relationship was observed between the Co and Fe content and the coercive field value. Numerous studies of co-authors have shown that elements such as Zr, Mo, W, or Nb [23,24] have a significant impact on the reduction of H_C_ in alloys based on Fe and Co. In addition, the authors observed a relationship between the value of the coercive field and the width of the amorphous halo. For a wider maximum, the alloy shows a lower coercive field value. The shape of the maximum can be related with a different arrangement of the alloys tested. Alloys characterised by a larger maximum width may have a structure with the best configuration of Fe and Co atoms, which determines the magnetic properties of these materials.

## 5. Conclusions

This paper presents the results of an investigationinto the magnetic properties of bulk alloys based on Fe and Co. All of the studied alloys have an amorphous structure. Based on the results, the following conclusions were drawn:

1. Fe addition, at the expense of Co, results in monotonic changes in magnetic parameters: decreases in T_C_ and D_spf_, and an increase in M_S_;
2. Higher content of Co in the tested alloys does not affect the reduction of H_C_ value;
3. In the volume of the studiedalloys, there are areas characterised by a different structural arrangement, as evidenced by: the shapes of the magnetic hysteresis loop, the increased value of magnetic saturation polarisation above the Curie temperature, and the decrease in the value of the D_spf_ parameter, despite the increase in saturation magnetisation;
4. All of the tested alloys are characterised by a similar quantity and nature of structural defects; the magnetisation process in the approach to ferromagnetic saturation area is associated with the rotation of the magnetisation vector around linear defects.

## Figures and Tables

**Table 1:** Data from the analysis of X-ray diffraction patterns.

Alloy	Width of the Maximum (deg)	Intensity of the Maximum (a.u.)	Position of the Maximum (deg)
Fe_36_Co_36_Y_8_B_20_	9.2	3600	42.77
Fe_39_Co_33_Y_8_B_20_	9.2	3300	43.15
Fe_43_Co_29_Y_8_B_20_	10.4	4000	42.98
Fe_48_Co_24_Y_8_B_20_	9.5	2800	42.97

**Table 2:** Magnetic properties of the tested alloys.

Alloy	Width of the Maximum (deg)	H_c_ (A/m)	M_s_ (T)	T_c_ (K)	b (10^−2^ T^1/2^)	A_ex_ (10^−12^ J/m)	D_spf_ (meVnm^2^)
Fe_36_Co_36_Y_8_B_20_	9.2	208	1.09	745	4.7	1.82	51
Fe_39_Co_33_Y_8_B_20_	9.2	169	1.1	720	4.8	1.81	50
Fe_43_Co_48_Y_8_B_20_	10.4	72	1.1	710	5.0	1.76	49
Fe_48_Co_24_Y_8_B_20_	9.5	75	1.15	675	5.2	1.72	48

H_C_—coercive field; M_S_—saturation magnetisation.
